# Management of children and adolescents with chronic myeloid leukemia in blast phase: International pediatric CML expert panel recommendations

**DOI:** 10.1038/s41375-023-01822-2

**Published:** 2023-01-27

**Authors:** Stephanie Sembill, Maria Ampatzidou, Sonali Chaudhury, Michael Dworzak, Krzysztof Kalwak, Axel Karow, Alexander Kiani, Manuela Krumbholz, Maaike Luesink, Nora Naumann-Bartsch, Barbara De Moerloose, Michael Osborn, Kirk R. Schultz, Petr Sedlacek, Fiorina Giona, Christian Michel Zwaan, Hiroyuki Shimada, Birgitta Versluijs, Frederic Millot, Nobuko Hijiya, Meinolf Suttorp, Markus Metzler

**Affiliations:** 1 Pediatric Oncology and Hematology, Department of Pediatrics and Adolescent Medicine, University Hospital Erlangen, Erlangen, Germany; 2 Comprehensive Cancer Center Erlangen-EMN (CCC ER-EMN), Erlangen, Germany; 3 Department of Pediatric Hematology-Oncology, Aghia Sophia Children’s Hospital, Athens, Greece; 4 Division of Pediatric Hematology/Oncology/Stem Cell Transplantation, Ann & Robert H. Lurie Children’s Hospital of Chicago, Northwestern University Feinberg School of Medicine, Chicago, IL USA; 5 St. Anna Kinderspital, Department of Pediatrics, Medical University, Vienna, Austria; 6 Department of Pediatric Hematology, Oncology and BMT, Wroclaw Medical University, Wroclaw, Poland; 7 Medizinische Klinik IV, Klinikum Bayreuth GmbH, Bayreuth, Germany; 8 Princess Máxima Center for Pediatric Oncology, Utrecht, the Netherlands; 9 Department of Pediatric Hematology-Oncology and Stem Cell Transplantation, Ghent University Hospital, Ghent, Belgium; 10 Women’s and Children’s Hospital and Royal Adelaide Hospital, Adelaide, SA Australia; 11 Division of Hematology/Oncology/BMT, British Columbia Children’s Hospital Research Institute, Vancouver, BC Canada; 12 Department of Pediatric Hematology and Oncology, University Hospital Motol, Prague, Czech Republic; 13 Department of Translational and Precision Medicine, Sapienza University of Rome, Rome, Italy; 14 Erasmus MC-Sophia Children’s Hospital, Rotterdam, the Netherlands; 15 ITCC Hematological Malignancies Committee, Rotterdam, the Netherlands; 16 Department of Pediatrics, Keio University School of Medicine, Tokyo, Japan; 17 Departments of Paediatric Oncology/Haematology, Poitiers University Hospital, Poitiers, France; 18 Division of Pediatric Hematology/Oncology/Transplant, Columbia University Irving Medical Center, New York, NY USA; 19 Pediatric Hemato-Oncology, Medical Faculty, Technical University Dresden, Dresden, Germany

**Keywords:** Myeloproliferative disease, Paediatrics, Chronic myeloid leukaemia

## Abstract

Treatment of chronic myeloid leukemia has improved significantly with the introduction of tyrosine kinase inhibitors (TKIs), and treatment guidelines based on numerous clinical trials are available for chronic phase disease. However for CML in the blast phase (CML-BP), prognosis remains poor and treatment options are much more limited. The spectrum of treatment strategies for children and adolescents with CML-BP has largely evolved empirically and includes treatment principles derived from adult CML-BP and pediatric acute leukemia. Given this heterogeneity of treatment approaches, we formed an international panel of pediatric CML experts to develop recommendations for consistent therapy in children and adolescents with this high-risk disease based on the current literature and national standards. Recommendations include detailed information on initial diagnosis and treatment monitoring, differentiation from Philadelphia-positive acute leukemia, subtype-specific selection of induction therapy, and combination with tyrosine kinase inhibitors. Given that allogeneic hematopoietic stem cell transplantation currently remains the primary curative intervention for CML-BP, we also provide recommendations for the timing of transplantation, donor and graft selection, selection of a conditioning regimen and prophylaxis for graft-versus-host disease, post-transplant TKI therapy, and management of molecular relapse. Management according to the treatment recommendations presented here is intended to provide the basis for the design of future prospective clinical trials to improve outcomes for this challenging disease.

## Introduction

Chronic myeloid leukemia (CML) blast phase (CML-BP) is characterized by clinical, phenotypic, and genetic features of acute leukemia. CML-BP can occur as an initial presentation of CML (*de novo* CML-BP) or as a progression from the chronic (CP) or accelerated (AP) phases of CML (secondary CML-BP).

After the introduction of tyrosine kinase inhibitors (TKIs), the annual rate of progression from CML-CP to AP or BP dramatically decreased to 1–1.5% from more than 20% [1, 2]. However, the prognosis for adult patients with *de novo* or secondary CML-BP remains poor; median survival is less than 1 year [3]. The low incidence of CML in pediatric patients (1–2.2 cases per million per year, with 7–10% being diagnosed with BP) and the lack of specific clinical trials mean that there is minimal evidence to inform the optimal management of CML-BP in children and adolescents [4]. In contrast to adult patients, children and adolescents with CML-BP present with a distinct predominance of the lymphoid phenotype (70–80% vs. 20–30% in adults) and a varying landscape of chromosomal aberrations differing from the classical major route additional cytogenetic aberrations in advanced phase adult CML [5–7]. Those leukemic features also complicate the distinction between pediatric *de novo* lymphoblastic CML-BP and Philadelphia chromosome-positive (Ph^+^) acute lymphoblastic leukemia (ALL), confronting treating physicians with further therapeutic uncertainty.

Thus, the treatment of CML-BP remains one of the major challenges in the management of patients with CML. Although the overall prognosis in CML-BP seems to be better in children than adults, consistent data regarding the optimal management of CML-BP in children and adolescents are lacking given small case numbers [6, 7]. Published evaluations of various cohorts are highly heterogeneous in their treatment regimens for *de novo* and secondary CML-BP in children.

The practical recommendation aims to reduce variability in the management of pediatric patients diagnosed with CML-BP, with particular focus on the diagnosis, treatment, and monitoring of this challenging disease stage. Efforts to standardize the approach may provide more consistent data and, potentially, a platform for future clinical studies.

### Composition of the expert panel and the consensus-building process

The panel of experts—22 members of the pediatric CML working party (pCML-WP) of the International-Berlin-Frankfurt-Münster (BFM) study group and additional national representatives, all with clinical and research expertise in pediatric CML and hematopoietic stem cell transplantation (HSCT)—met annually at the I-BFM plenary meetings and at additional regular online working meetings. Unless otherwise indicated, there was complete consensus for the recommendation after the panel discussion. The panel discussion was based on a systematic search of the English-language literature in the Cochrane Library, PubMed, and Scopus databases. The search criteria specified studies involving pediatric patients with CML in an advanced phase and treatment with HSCT, using these terms: (“chronic myeloid leukemia” OR “CML”) AND (“pediatric” OR “children” OR “adolescents” OR “childhood”) AND (“advanced phase” OR “accelerated phase” OR “blast phase”). Searches were conducted on January 18, 2022, and were limited to studies published from January 1, 2000, to December 31, 2021. The resulting 104 unique records were screened by title and abstract. Case reports or case series with fewer than 10 patients and studies in adult patients or studies that excluded advanced phases of CML were excluded. The 19 resulting articles underwent a full-text assessment for eligibility, which finally identified 10 studies that provided specific data about HSCT in pediatric patients with CML-BP (Table 1). The resulting recommendation is based on these pediatric studies and, when no such data were available, on recommendations adapted from those for adult patients and from experience in pediatric Ph^+^ ALL. Evidence was graded according to the GRADE (Grading of Recommendations Assessment, Development and Evaluation) system. The grade of recommendation for each statement is summarized in Supplementary Table 1.

**Table 1:** Overview of retro- and prospective studies in the TKI era including pediatric CML patients in BP.

Study	Design	period	No. entire cohort	No. SCT	Donor type	Conditioning regimen	Stem Cell Source	Prior TKI therapy	Prior CT advanced phase	Disease status at transplant reported	Post-transplant TKI	GVHD	Outcome/Endpoints
Sembill et al. [6]	r, m	2007–2020	BP (18)	BP (14)	MUD (8) MRD (2) MMD (2) no data (2)	TBI-based (5) Bu-based (4) others (3) no data (2)	n.a.	Y (12) N (2)	Y (12) no data (2)	Y	n.a.	GvHD (7)	OS
Hafez et al. [60]	r, s	2007–2017	CML (43) BP (4)	CML (43) BP (4)	MRD (43)	Bu based (43)	BM (32) PBSC (11)	Y (37) N (6)	Y (4)	Y	Y (4)	aGvHD (5) cGvHD (19)	OS EFS TRM
Meyran et al. [5]	r/p, s	2001–2016	CML (339) Adv. P (19)	Adv. P (16)	MUD (7) MRD (8) MMD (1)	TBI-based (7) Bu-based (3) no data (6)	BM (10) PBSC (3) CB (2) No data (1)	Y (16)	Y (13) N (3)	Y	Y (8)	n.a.	CHR, CCyR, MMR, OS
Millot et al. [7]	r/p, m	2000–2017	CML (479) AP (19) BP (17)	HSCT (17) AP (6) BP (11)	MRD (8) MUD (7) MMD (2)	n.a.	n.a.	Y (17)	Y (13) N (4)	Y	n.a.	n.a.	CHR, CCyR, MMR, OS
Suttorp et al. [24]	p, m	2004–2017	CML (156) AP (3) BP (7)	CML (28) AP (1) BP (4)	n.a.	n.a.	n.a.	n.a.	n.a.	n.a.	n.a.	n.a.	CHR, CCyR, MMR, OS
Shulman et al. [67]	r, s	2010–2013	Adv. P (5) AP (1) BC (4)	Adv. P (5) AP (1) BC (4)	MRD (1) MUD (4)	TBI based (5)	BM (5)	Y (5)	Y (4) N (1)	Y	Y	aGvHD (1) cGvHD (1)	OS MMR
Suttorp et al. [58]	p, m	1995–2004	CML (200) AP (17) BP (14)	CML (176) AP (9) BP (9)	MRD (41) MUD (71) MMD (55)	Bu based (79) TBI-based (82) Others (15)	BM (102) PBSC (64) CB (2)	n.a.	Y	n.a.	n.a.	aGvHD (62) cGvHD (36)	TRM OS
Muramatsu et al. 2010	r, m	1993–2005	CML (125) Adv. P. (37)	CML (125) Adv. P (37)	UD (125) MRD (41)	TBI-based (96) Others (29)	BM (125)	Y (17)	n.a.	Y	n.a.	aGvHD (77) cGvHD (51)	OS TRM LFS
Unal et al. 2007	r, s	1997–2006	CML (14) AP (3)	CML (14) AP (3)	MRD (12) MUD (2)	Bu based (13) Others (1)	BM (14)	Y (3) N (11)	n.a.	n.a.	n.a.	aGvHD (1) cGvHD (1)	OS EFS
Millot et al. 2003	r. m	1982–1998	CML (76) Adv. P (29)	CML (76) Adv. P (29)	MRD (60) UD (16)	TBI-based (53) Bu based (20) others (3)	n.a.	n.a.	n.a.	n.a.	n.a.	aGvHD (6) cGvHD (3)	OS EFS

*Adv. P* advanced phase, *aGvHD* acute GvHD, *AP* accelerated phase, *BM* bone marrow, *BP* blast-phase, *Bu* Busulfan, *CB* cord blood, *CCyR* complete cytogenetic remission, *cGvHD* chronic GvHD, *CHR* complete hematologic remission, *EFS* event free survival, *Y* Yes, *LFS* leukemia free survival, *m* multicenter, *MMD* mismatched donor, *MMR* major molecular response, *MRD* matched related donor, *MUD* matched unrelated donor, *n.a.* not available, *N* No, *OS* overall survival, *p* prospective, *PBSC* peripheral blood stem cells, *r* retrospective, *s* single-center, *TBI* total body irradiation, *TRM* treatment related mortality, *UD* unrelated donor.

## Recommendations

### Diagnosis of CML-BP and patients at risk for progression

Accurate diagnosis of suspected CML-BP is critical for further therapeutic steps [8, 9]. The definitions of CML-BP are not consistent across international committees. The European LeukemiaNet defines CML-BP as 30% or more blast cells in blood or bone marrow and/or the demonstration of extramedullary blastic infiltrates with the exception of the liver and spleen [10]. The World Health Organization and recent International Consensus Classification (ICC) classification sets the threshold value for blasts at 20% [11, 12]. Furthermore, in the current WHO classification, CML-AP is no longer a diagnostic category and growing importance is given to patients at risk for progression based on somatic mutations, TKI resistant *BCR::ABL1* mutations and/or additional chromosomal aberrations (ACAs). It is also emphasized that the detection of lymphoblasts in peripheral blood or bone marrow, even in the range below 10%, is generally considered consistent with the diagnosis of blast phase [11]. The ICC similarly states that increasing numbers of lymphoblasts (>5%) in peripheral blood or bone marrow may indicate impending lymphoid BP and should prompt further investigations [12]. A comparison of the World Health Organization classification system with the 20% cut-off used in the imatinib trials showed that the response rate was significantly better in adult patients with 20–29% blasts than those with 30% or more [13]. Current pediatric guidelines recommend classification according to ELN criteria. [8, 14]. However, the above-mentioned additional genetic features should also be included in pediatric CML for improved diagnostics.

In patients who meet the criteria of CML-BP, the morphologic diagnosis should be accompanied by immunophenotyping to characterize the blast population. The blasts in CML-BP can be of the myeloid, lymphoid, or mixed-lineage phenotypes [14]. Lymphoid blasts are in most cases derived from the B cell lineage; only very rare cases with a T cell origin have been described [15, 16]. After the cytogenetic detection of the t(9;22) translocation, the next step is to identify the transcript type. In CML, most breakpoints cluster in the major breakpoint region, encoding for the p210^BCR-ABL1^ fusion protein. More than 90% of patients harbor either the e13a2 or e14a2 fusion transcript or, in cases of alternative splicing, both transcripts [17]. Additionally, several atypical rare *BCR::ABL1* fusion variants have been detected [17, 18]. A minor breakpoint encoding for the p190^BCR-ABL1^ fusion protein has been identified in only a few cases. As in adult advanced phases, a recent study documented that two-thirds of pediatric patients harbor one or two *BCR::ABL1* tyrosine kinase domain mutations at diagnosis (*de novo* CML-BP 75%, secondary CML-BP 62%) [6]. Therefore, performing an analysis for *BCR::ABL1* mutations is strongly recommended, as the selection of a sensitive TKI is crucial for individualized therapy planning. Next-generation sequencing (NGS) is preferred over Sanger sequencing as the higher sensitivity (3% vs. 20% threshold) allows the early detection of relevant resistance mediating mutations [19].

A final major challenge is to detect and differentiate *de novo* CML-BP from Ph^+^ acute leukemia. If *de novo* CML-BP presents as an acute myeloid neoplasm, an assignment based on cytogenetics or molecular genetics by detection of the t(9;22) translocation or the *BCR::ABL1* fusion transcript becomes obvious, because acute myeloid leukemia (AML) with *BCR::ABL1* is a rare entity. In the AML-BFM trials, only six children with Ph^+^ AML were diagnosed among a total cohort of nearly 1500 patients with AML (age range: 1–18 years) over a period of 16 years (Dirk Reinhardt, AML-BFM study chair, database query January 1, 2022), and it is unclear if single cases could not also have been designated as myeloid BP [20]. However, in 2016, the World Health Organization added AML harboring *BCR::ABL1* as a new category of AML that might benefit from TKI therapy [21]. In case of difficulties in the diagnostic distinction between entities, certain clinical, cytogenetic, and molecular genetic features can support the diagnosis of CML-BP [22, 23].

#### pCML-WP recommendation

An extended diagnostic workup should be completed for pediatric patients who meet the criteria for CML-BP (Table 2). This should always include *BCR::ABL1* mutation testing by NGS.

**Table 2:** Baseline diagnostics of suspected BP in pediatric patients.

Test at diagnosis of BP	
Physical examination	In particular spleen and liver size [cm below the costal margin], extramedullary manifestation of disease
Complete Blood Count	Full white blood cell (WBC) count Hemoglobin (Hb) and platelet count, neutrophils, lymphocytes, monocytes, Basophils %, Eosinophils %, Blasts %, Sum of Blasts % + Promyelocytes %
Bone Marrow	Morphology Blast % and Promyelocytes % Cytogenetics (for Ph-positive and/or additional chromosomal aberrations) with a minimum of 15 metaphases analyzed Fluorescent-in-situ-hybridization (FISH) if marrow cytogenetics fails Trephine biopsy (assessment of focal “nests of blasts” and fibrosis
Flow cytometry and/or cytochemistry	Definition of phenotype by expression of cell-surface and cytoplasmic markers (myeloid vs. lymphoid)
Molecular genetics	*BCR::ABL1* fusion transcript (e13a2, e14a2, e1a2 or any rare type) *BCR::ABL1* transcript level quantitative analysis (on the IS scale) Tyrosine kinase domain mutation analysis NGS myeloid or lymphoid panel
Cerebrospinal Fluid	Cytology Intrathecal injection for CNS prophylaxis (for details see text)
Human leukocyte antigen (HLA)-typing	Donor search for allogeneic stem cell transplantation

### Discriminating Ph^+^ ALL from CML-BP lymphoid phenotype

Most children and adolescents with *de novo* CML-BP present with lymphoid blasts, leading to diagnostic uncertainty in discriminating that entity from Ph^+^ ALL [5–7, 24]. Certain morphological criteria, such as increased numbers of basophils or eosinophils when patients present with less than 70% blast cells in bone marrow, could be indicative of CML-BP rather than Ph^+^ ALL. Leukocytosis with left-shifted myeloid maturation might also point toward a diagnosis of CML-BP [25]. Current studies have shown that pediatric patients with *de novo* CML-BP often present with high leukocyte counts at the time of diagnosis [6, 7]. The presence of the p210^BCR-ABL1^ fusion protein is likewise suggestive but has also been observed in 10–20% of Ph^+^ ALL cases [26]. Other molecular markers, such as *IZKF1* deletions, also occur in both entities and are not solely characteristic of Ph^+^ ALL [27, 28]. In most cases, a valid distinction between the entities based on morphology, immunophenotyping, molecular genetics, and transcript type at the time of diagnosis is impossible. However, indicators to better discriminate them can emerge during therapy. Typical Ph^+^ ALL affects the lymphoid lineage only; in CML-BP lymphoid phenotype, the *BCR::ABL1* rearrangement is present in both the lymphoblastic and expanded myeloid cell clones. Therefore, at the end of an ALL induction in CML-BP, a discrepancy is often observed between low minimal residual disease (MRD) as assessed by immunoglobulin G heavy chain (IgH) or T cell receptor gene (TCR) rearrangement markers or flow cytometry (because of a preferential reduction in the lymphoid blast clone) and the still relatively high proportion of t(9;22) translocation–positive cells (of the still expanded *BCR*::*ABL1-*positive hematopoiesis) in florescence in situ hybridization (FISH) or transcript analysis [29]. This discordant MRD dynamic is also described as a feature of the subset of Ph^+^ ALL designated as CML-like disease [30, 31]. Polymerase chain reaction (PCR) analysis of sorted cells does not yield reliable results because of the adverse balance between exponential amplification in PCR and the purity of the cell populations sorted from clinical samples. In principle, interphase FISH on neutrophils might expose the *BCR::ABL1* rearrangement on cells of myeloid origin after a major blast reduction has occurred—for example, after two weeks of ALL-based induction treatment [32, 33]. The limitations of that method are a lack of universal availability of the test or the achievement of a remission that is already too deep to detect the *BCR::ABL1* fusion by FISH. With respect to test availability, dried, unstained bone marrow smears stored frozen (−18 °C) can be wrapped in aluminum foil and sent to specialized laboratories. However, even with those extended diagnostic procedures (Table 3), a definitive distinction can remain elusive in some patients.

**Table 3:** Diagnostics for differentiation of de-novo lymphoid BP and Ph-positive ALL.

Investigation	de-novo lymphoid BP	Ph-positive ALL	Comment
Morphology	=	=	
Flow cytometry	=	=	
Cytogenetics/FISH	=	=	Cytogenetic or FISH positivity at the end of induction therapy as an indicator for CML-BP
*BCR::ABL1* detection via interphase FISH on neutrophils	+	−	performed after major blast reduction
Comparison MRD-level (IgG, TCR -rearrangement or flow cytometry)/ *BCR::ABL1* transcript level	↓/~	↓/↓	performed after the end of induction therapy

= indifferent, + positive result, - negative result, ↓ declining, ~ divergent reduction.

#### pCML-WP recommendation

To date, no single marker unambiguously differentiates *de novo* CML-BP from Ph^+^ ALL in all cases at the time of diagnosis. In such cases, additional parameters must be obtained after therapy commences (Table 3). A high leukocyte count, left-shifted myeloid maturation, or an increased basophil or eosinophil count at diagnosis and the presence of a major transcript type are indicative, but not conclusive, in diagnosing *de novo* CML-BP. The panel, therefore, recommends using real-time PCR for *BCR::ABL1* and IgH/TCR or flow cytometry markers (if identifiable at diagnosis) in parallel to monitor response to therapy. The accuracy of diagnosis should then be reevaluated as therapy proceeds if a divergence between the clonal markers and *BCR::ABL1* transcript levels is detected. The clear detection of *BCR::ABL1* in neutrophils by interphase FISH is confirmatory for CML-BP.

### Treatment principles in CML-BP

The general aim of curative therapy in adult patients is to reduce the blast population to reach a CP and to proceed with allogeneic HSCT [34, 35]. Selection of the optimal patient-specific therapeutic pathway depends on the BP phenotype, the presence of *BCR*::*ABL1* kinase domain mutations, previous therapy for secondary CML-BP, and the availability of suitable stem cell donors. The rationale for HSCT is that, in the pre-TKI era, intensive chemotherapy alone without consolidative HSCT produced only a short response with a high relapse rate in CML-BP. However, even with TKI therapy, the response is usually only temporary.

In principle, clinical, cytogenetic, and molecular genetic risk factors can support therapy stratification. Clinical parameters such as older age, low red cell and platelet counts, high lactate dehydrogenase, myeloid immunophenotype, and other features associated with poorer survival or higher risk of treatment failure that can be systematically evaluated in adults have no known correlates in children because of smaller case numbers [13, 36]. Risk scores such as the EUTOS long-term survival score do not play a role in prognostic assessment once progression to CML-BP has occurred, neither in adult nor in pediatric patients. Cytogenetic risk factors from adult studies do not apply to childhood CML, because the cytogenetic profile of young patients differs significantly from that of older patients. A recent analysis of cytogenetics in pediatric CML-BP showed that complex karyotypes were more common in patients with secondary CML-BP than in patients with de novo CML-BP; however, risk factors within those subgroups could not be conclusively identified [6].

Somatic mutations in genes *RUNX1*, *TP53*, *ASXL1*, and *WT1*, which also occur in other myeloid malignancies, have been found with a high prevalence in adult patients with CML-BP. The presence of these mutations at diagnosis is associated with high risk of disease progression in CML. A recent comprehensive genetic analysis demonstrated that, compared with clinical parameters, specific genetic lesions are better predictors of survival in CML-BP [37, 38]. Only a small case series on somatic mutations in childhood CML-CP has been published, in which only *ASXL1* mutations were identified in 6 of 21 patients with CML [39]. Those prognostic markers currently play no role in therapy selection in pediatric CML-BP.

To achieve sustained remission, the current treatment options for adult patients with adequate performance status include lineage-specific induction chemotherapy in combination with a high-potency TKI [40]. The same concept has been applied in most pediatric cases published to date. The established ALL and AML induction regimens for the specific BP immunophenotype are suitable [5–7]. Although a few cases showing a response to TKI monotherapy have been documented, chemotherapy in combination with a TKI is the cornerstone, considering the risk of resistance to TKI monotherapy and the high relapse rates in CML-BP [6, 7]. Until the diagnosis is confirmed by the detection of *BCR::ABL1* and the definition of the immunophenotype (lymphoid vs. myeloid), supportive therapy is administered as in acute leukemia (prevention of tumor lysis syndrome according to institutional standards). Hydroxyurea could play a role in clinically stable patients if a definitive diagnosis cannot be established within a short period. In patients with signs and symptoms of leukostasis, lineage-adapted cytoreduction measures according to ALL or AML protocols are recommended.

#### pCML-WP recommendation

Allogeneic HSCT is strongly recommended for most children with CML-BP. Beforehand, a phenotype lineage-appropriate induction therapy in combination with a TKI should be administered.

### Selection of TKI therapy

The selection of TKI therapy is challenging, and data for efficacy in CML-BP are limited. The selection of the TKI depends on the BP type (*de novo* vs. secondary) and the presence of *BCR::ABL1* kinase domain mutations. Currently, no second-generation TKI (2G-TKI) is approved for pediatric patients in CML-BP; however, imatinib and dasatinib have approval for use in Ph^+^ - ALL. For this reason, off-label use will be necessary in most cases but is considered justified given the risk of the condition.

*Selection of TKI for secondary CML-BP*. In secondary CML-BP that has developed during TKI therapy, it is strongly recommended to switch the TKI. Approximately 60% of pediatric patients with secondary CML-BP showed mutations in the tyrosine kinase domain of the *BCR::ABL1* gene [6]; the TKI must therefore be selected according to the tyrosine kinase domain mutation profile. If secondary CML-BP develops after treatment with a 2G-TKI, or if a T315I mutation is present, ponatinib is the appropriate TKI. However, caution must be taken with increased toxicity of ponatinib with intensive chemotherapy. For example, ponatinib and asparaginase have overlapping toxicities (e.g., pancreatitis and hepatotoxicity) which may preclude concurrent use of these drugs. Ponatinib use in children, both as a single agent (NCT03934372) and in combination with chemotherapy (NCT04501614), is currently under investigation.

*Selection of TKI for de-novo CML-BP*. The faster achievement of a deep remission as a basis for stem cell transplantation is the rationale for the frontline use of a 2G-TKI in *de novo* CML-BP. In *de novo* BP, a tyrosine kinase domain mutation analysis should be performed at diagnosis, because a resistance-mediating mutation is already present in a large proportion of cases, affecting selection of the optimal TKI [6, 7]. Resistance may also occur later and hence repeated screening may be needed. In cases of inadequate response to therapy with 2G-TKI and induction therapy, a switch to ponatinib should also be considered to not delay HSCT. The exact integration of TKI treatment into the respective leukemia protocols is outlined in the next subsections.

#### pCML-WP recommendation

We recommend the upfront use of a 2G-TKI in *de novo* CML-BP and if secondary BP has developed following treatment with imatinib. The presence of a resistance-mediating *BCR::ABL1* kinase domain mutation should guide the appropriate TKI selection. Switching to ponatinib is performed in the presence of a T315I mutation, but should also be considered in the absence of *BCR::ABL1* kinase domain mutations if second-line therapy fails.

### Treatment of CML-BP lymphoid phenotype

In adult patients, the preferred curative approach combines the use of lineage-specific induction chemotherapy with a TKI [34, 35]. Therapeutic approaches with documented tolerable toxicities that can be combined with TKI therapy include hyperCVAD and Ida-FLAG [41–43]. Steroids combined with a TKI is recommended by NCCN for adult patients who cannot tolerate standard induction chemotherapy [40].

In pediatric patients, multi-drug induction therapy combined with a TKI has been studied in multiple trials in Ph^+^ ALL. In evaluations of imatinib or dasatinib combined with standard chemotherapy regimens, toxicities were found to be tolerable [44–46]. A reduced induction approach such as combination of steroid and vincristine is used for adults with CML-BP to reduce toxicities related to advanced age and concomitant diseases. Such regimens, in combination with a TKI, can often achieve remission at the end of induction, even with less intensive induction regimen (without anthracyclines) or shortened duration (e.g., only until day 15 of induction in good responders). Bone marrow aspiration on day 15 may be used to assess treatment response. If the blast percentage is sufficiently low (for instance, <5%), chemotherapy may be terminated, and TKI alone may be continued. If the response is not optimal on day 15, the second half of the induction chemotherapy (with anthracyclines) should be used for intensification. The reasoning in favor of full ALL induction therapy in pediatric patients is based on the advantages of a deeper remission and a better chance for early transplantation with a low MRD in patients with ALL and an acceptable toxicity profile for induction therapy in combination with a TKI.

No complete consensus could be reached for the recommended induction therapy for children and adolescents with lymphoid CML-BP phenotype. The positions taken by national study groups range from complete induction therapy as used in ALL, to reduced induction with only a part of the multi-agent regimen, depending on the response. Because no formal evidence for the superiority of either approach has been developed, mandating either option without the full agreement of the pCML-WP would pose more of a risk for therapy management in this critical entity. The pCML-WP’s preliminary agreement was that, until a better database is available, the regimen that accords with national and individual experience would be recommended.

The second topic of discussion relating to this issue was the timing of TKI initiation: immediately on initiation of induction therapy, or starting on day 15 as in the EsPhALL protocol for *BCR::ABL1-*positive ALL. There was consensus within the expert panel that therapy should start immediately upon confirmation of the presence of *BCR::ABL1*. Dose reduction or discontinuation should be considered in cases of prolonged aplasia after induction treatment. The response to therapy should be assessed in the same way as in acute leukemia, following the standards of the respective protocol for morphology, cytogenetics, flow cytometry, and molecular MRD markers including *BCR::ABL 1* transcripts by real-time PCR and IgH/TCR markers.

The parallel measurement of clonal MRD markers and *BCR::ABL1* transcripts can provide new insights into the response of the blast population in patients with CML-BP lymphoid phenotype. However, the ideal depth of remission at the end of induction therapy has not yet been systematically studied in this patient group. Intensification of chemotherapy or implementation of alternative therapy concepts in the rare cases of hematologic non-response in bone marrow at the end of induction or of the lack of a suitable donor for allogeneic HSCT should be discussed case-by-case.

Intrathecal chemoprophylaxis is as important for pediatric patients with CML-BP lymphoid phenotype as it is for those with acute leukemia. Compared with imatinib, dasatinib has better penetration into the cerebrospinal fluid (CSF); however, no TKI reaches a sufficient concentration in the CSF to safely treat and prevent central nervous system (CNS) disease [47]. Isolated CNS relapse has been described in adult patients treated for CML-BP lymphoid phenotype [48, 49]. There are several cases of lymphoid blast crisis with CNS involvement in the pediatric CMLpaed II registry. The optimal number and choice of drugs remain unclear. Until better data are available, the expert panel recommends a prophylactic approach meeting the standards of the induction chemotherapy protocol for patients with CNS 1 or 2 status. Prophylactic intrathecal administration should continue monthly as bridging therapy until transplantation. Presentation in CNS-status 3 with infiltration of blasts into the CSF (>5/µl) is particularly rare; only a few adult cases have been reported. The benefits of cranial irradiation in children and adolescents with CML-BP lymphoid phenotype with CNS involvement are unclear. Some adult patients with CNS-status 3 have been treated with radiation therapy [49, 50]; however, no evidence-based therapy recommendations can be derived from those reports. The pCML-WP suggests that patients with CNS-positive status at diagnosis be treated with intrathecal chemotherapy according to the institution’s standards. Further treatment then depends on the timing of the HSCT.

For patients with Ph^+^ ALL, the EsPhALL protocol recommends a cranial boost before total body irradiation (TBI) in patients with CNS involvement. Whether adopting this concept for CML-BP lymphoid phenotype would be beneficial cannot be assessed; the patient numbers are too small, and data are lacking. In cases of CNS involvement, the panel recommends a TBI-based conditioning regimen with a cranial boost.

#### pCML-WP recommendation

Initial therapy is recommended to follow the institutional standard for ALL induction therapy in combination with a TKI. Prophylactic intrathecal therapy is mandatory.

### Treatment of CML-BP myeloid phenotype

Myeloid BP confers a worse prognosis than lymphoid BP in adult CML [38]. Intensified therapies are therefore used in adult patients to induce a hematologic response before HSCT. In the AFR01 study, Deau et al. investigated imatinib and cytarabine combined with daunorubicin (“3 + 7” regimen) and reported higher rates of complete hematologic remission with the addition of daunorubicin [51]. The combination of ponatinib with fludarabine, cytarabine, and idarubicin was investigated in the MATCHPOINT trial [43]. No prognostic difference can be determined according to the immunophenotype for children and adolescents because of the small number of cases.

Current studies in pediatric patients with CML-BP myeloid show that AML induction combined with a TKI is used in the majority of cases [5–7]. However, systematic studies on tolerability in this patient group are lacking. For all pediatric patients with CML-BP myeloid phenotype (*de novo* and secondary), the pCML-WP recommends implementing the first AML induction block following national standards. The response criteria that should be met for CML-BP myeloid phenotype after the first induction element have not yet been defined. Unnecessary toxicity from additional chemotherapy meant only to achieve a lower MRD in morphological complete remission before transplantation should be avoided. Moreover, in CML-BP myeloid phenotype, TKIs are available as supplemental therapeutic agents. Given the lack of data, the pCML-WP recommends following the response parameters used in pediatric *de novo* AML protocols. Whether a second cycle of chemotherapy should be added depends on remission status and the availability of a suitable donor.

Based on the experience in adult patients and pediatric AML studies, the panel’s recommendation is to start TKI therapy not in parallel with chemotherapy, but directly at the end of induction. This approach helps to avoid interactions during chemotherapy. Whether it also results in reduced toxicity is not yet clear.

Not all adult protocols recommend prophylactic intrathecal treatment. In pediatrics, the panel recommends following the recommendations of the AML induction protocol and administering the prescribed CNS prophylaxis. In cases with CNS-status 3, individualized treatment decisions are necessary, with most clinicians administering at least weekly intrathecal chemotherapy until clearance of blasts.

#### pCML-WP recommendation

To induce hematologic remission in CML-BP myeloid phenotype, a course of AML induction according to the national and institutional standards is recommended. TKI therapy should be administered not in parallel with chemotherapy, but at the end of induction to avoid excessive toxicity and interactions. Intrathecal prophylaxis should be included according to national protocols.

### Treatment of CML-BP mixed phenotype

Specific data for CML-BP of ambiguous lineage are lacking. The proposed treatment strategy is therefore based on the pediatric data in acute leukemia. In a retrospective multinational trial, Hrusak et al. reported superior survival with ALL-type treatment in patients with ambiguous lineage acute leukemia [52]. Other therapy strategies are recommended only in the few cases with CD19 negativity and no other lymphoid features.

#### pCML-WP recommendation

The treatment strategy should be a combination of ALL induction therapy with a TKI unless other characteristics indicate a different therapeutic option.

### Timing of allogeneic HSCT in pediatric CML-BP

Allogeneic HSCT currently remains the only curative treatment option for most patients with CML-BP. Thus far, transplantation in active CML-BP has been identified as the strongest factor associated with poor outcome, and based on present knowledge, upfront HSCT without initial chemotherapy plays no role. The challenge in CML-BP is to balance the risk of progression during treatment against the benefit of achieving the lowest possible MRD before allogeneic HSCT.

In adults with CML-BP, the response to TKI treatment is very short-lived, and resistance mechanisms independent of *BCR::ABL1* often drive progression [53]. Thus, the general principle of reducing the *BCR::ABL1-*positive cell pool to its minimum over a prolonged period, as applied in CML-CP, is no longer transferable once CML has progressed to BP. However, Chen et al. demonstrated that residual disease in CML-BP, unlike that in CML-CP, not only has to drop below the threshold of a complete cytogenetic remission to achieve an acceptable prognosis but that five-year overall survival (OS) directly correlates with the depth of the molecular response (five-year OS in complete cytogenetic remission, 12%; in major molecular remission, 34%; in undetectable leukemia, 72%) [54]. Nevertheless, the presence of advanced disease at HSCT plays a crucial role in patient outcomes [36, 55]. Radujkovic et al. investigated 170 patients with CML-BP who were allografted after TKI pretreatment, finding that active BP was the factor most strongly associated with decreased OS [56].

In children and adolescents with CML-BP, current data demonstrated a high relapse rate of 15–27% after a median of 8.5 months (range: 6–15 months) and before HSCT could be performed [6]. Those results support an early allogeneic HSCT approach. A systematic analysis of the pre-HSCT variables associated with better outcomes has never been performed because the number of pediatric patients has been too low. The ideal timing for allogeneic HSCT depends on three factors: blast reduction, reduction in *BCR::ABL1* transcripts, and availability of a suitable stem cell donor. The current studies demonstrate that most pediatric patients achieve complete cytogenetic remission and a *BCR::ABL1* transcript level below 1% after acute leukemia induction chemotherapy with added TKI treatment [5–7].

No systematic data have yet been developed to assess the ideal depth of remission before transplantation based on clonal IgH/TCR rearrangement or flow cytometry for CML-BP. The option of deeper remission through consolidation therapy must always be weighed against the increased risk of relapse with delayed transplantation.

**Fig. 1:**
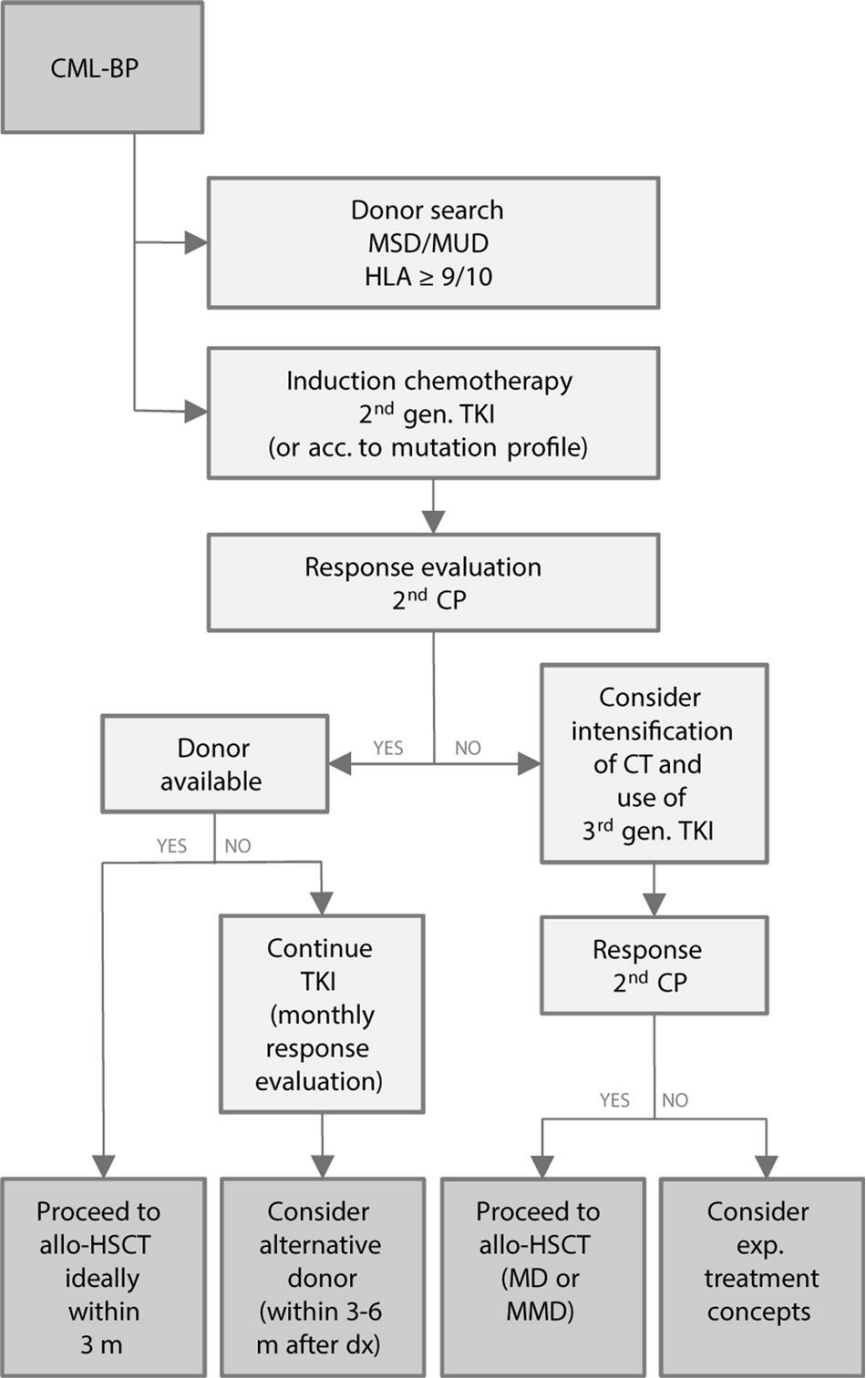
Timing of allogeneic hematopoietic stem cell transplantation (HSCT). acc. according, CT chemotherapy, dx diagnosis, gen. generation, m month.

The pCML-WP’s recommendation is therefore based on data from adults and limited experience in pediatric patients. Because of the high rate of early disease relapse, allogeneic HSCT should be initiated as early as possible after hematologic remission has been achieved (second CP). Figure 1 contains practical guidance about the timing of transplantation depending on the response to therapy and availability of a suitable donor.

#### pCML-WP recommendation

Allogeneic HSCT should be performed as soon as possible after, as a minimal requirement, a hematologic remission (second CP) has been achieved, ideally within 3 months.

### Donor and graft selection

Relevant evidence on the impact of donor type in pediatric patients allografted for CML-BP in terms of engraftment benefits or survival is not yet available. However, the prospective multicenter study for children with ALL (ALL-SCT BFM-2003 Trial) demonstrated no differences in overall survival and event-free survival between matched sibling donors (MSD) and HLA-matched unrelated donors (MUD) compatible in at least 9 of 10 HLA loci. Significant benefits in engraftment were observed in MSD bone marrow transplantation [57]. Most studies in CML-CP showed a survival benefit for MSD; the limitation is that those are either retrospective studies and/or were conducted before there were significant improvements in HLA typing, donor search, and treatment of acute graft-versus-host disease (GvHD) [58–60]. In addition, these studies have mostly demonstrated a survival advantage for bone marrow as a stem cell source [59, 61].

The efficacy of umbilical cord blood as an alternative stem cell source for adult patients has been described, but a study in 74 adolescent and young adult patients, including 16 with CML-BP, showed that outcomes including acute GvHD, transplantation-related mortality, relapse, and long-term survival were comparable whether umbilical cord blood or peripheral blood stem cells or bone marrow from siblings were used [62]. Thus, umbilical cord blood is an alternative stem cell source. However, the yield of stem cells is often limited, and donor lymphocyte infusion (DLI) is usually not possible.

Haploidentical stem cell transplantation with post-transplant cyclophosphamide in children and adolescents with high-risk leukemia, including a small number with CML-BP, was associated with tolerable toxicity and OS rates in the 40–70% range [63–65]. Haploidentical HSCT in the absence of a suitably matched donor should therefore be considered for patients with CML-BP. The role of haploidentical transplantation is being tested in trials, with results pending at the time of writing.

#### pCML-WP recommendation

The recommended donor in allogeneic HSCT is a MSD or a MUD compatible in at least 9 of 10 HLA loci. To avoid delay in transplantation, alternative stem cell sources and donor types should be considered if no matched donor is available. As in pediatric high-risk leukemia, bone marrow from sibling donors is the preferred stem cell source. Nevertheless, the choice of the optimal donor and stem cell source remains an individual decision based on the preferences of the transplant center.

### Selection of a conditioning regimen and prophylaxis for GvHD

In advanced phases of CML in adult patients, intensive conditioning is generally recommended as offering the best chance of leukemia-free survival [36, 66]. To mitigate transplant-related toxicity, reduced-intensity conditioning (RIC) is a possible alternative.

In pediatric patients, experience with RIC comes predominantly from those with CML-CP. A retrospective study from Japan described long-term outcomes in 180 children, adolescents, and young adults with CML who underwent allogeneic HSCT between 2001 and 2014. Of 42 patients who received RIC, 4 were diagnosed in CML-BP, and 5 in CML-AP. When major cytogenetic remission was accomplished before HSCT in either phase, no difference in 5-year OS between a myeloablative conditioning regimen and RIC was observed [61]. The authors proposed RIC as a possible alternative for patients in AP or BP who achieve a good response to therapy before transplantation, but given the still small number of cases, the pCML-WP does not yet see the possibility of a general recommendation for RIC. Additionally, in children and adolescents, toxicity plays a lesser role, and the panel’s recommendation is therefore myeloablative conditioning because it offers the best chance of cure.

Most pediatric patients allografted for CML-CP receive a busulfan- or TBI-based conditioning regimen. No significant differences between the conditioning regimens have been observed [5–7, 24, 58, 60, 67]. Again, no specific data for children and adolescents with CML-BP are available. In children with high-risk ALL, the multinational randomized ALL SCTped 2012 FORUM trial found improved outcomes after TBI plus etoposide conditioning compared with chemotherapy conditioning [68].

Based on those results, the panel preferred TBI-based conditioning for patients more than 4 years of age with CML-BP lymphoid phenotype. No data are available for children younger than 4 years. However, such cases are extremely rare and must be assessed on an individual basis. For CML-BP myeloid phenotype, a non-TBI myeloablative conditioning regimen based on national or institutional treatment protocols is suggested.

For all patients with CNS positivity, regardless of disease phenotype, a TBI-based conditioning regimen with CNS boost according to institutional standards is recommended.

The impact of the graft-versus-leukemia effect in CML-CP cannot be extrapolated to CML-BP [69]. Recent studies in adults allografted in an advanced phase of CML showed no significant differences in OS and leukemia-free survival whether they did or did not have chronic GvHD [55, 56]. These patients are usually not as intensively pre-treated, and thus their graft failure rate might therefore be higher. In children, prioritizing GvHD prevention is suggested when a conditioning regimen is being devised. In the ideal scenario in which the patient achieves good remission before transplantation, GvHD prophylaxis with anti-thymocyte globulin, methotrexate, and cyclosporine is recommended whatever the graft source.

#### pCML-WP recommendation

Myeloablative conditioning is recommended. The choice of conditioning regimen is lineage dependent, with TBI-based conditioning being preferred for CML-BP lymphoid phenotype and any patient with CNS3 disease. GvHD prophylaxis is of particular importance in young patients and should include anti-thymocyte globulin. Post-transplant immunosuppressive therapy should be reduced as soon as possible.

### Post-transplant *BCR::ABL1* transcript monitoring

Post-transplant monitoring for acute leukemia is typically based on quantification of chimerism, disease-specific molecular markers, and/or clone-specific IgH/TCR rearrangements (when the blast origin is lymphoid). The discussion that follows addresses recommendations for surveillance of the *BCR::ABL1* translocation.

The assessment by reverse transcriptase quantitative PCR from peripheral blood specimens provides the advantage of close monitoring. Such monitoring is strongly indicated, especially early after allogeneic HSCT, because studies show that early appearance of the *BCR::ABL1* translocation is associated with significantly reduced relapse-free survival [70]. In the German pediatric CML-BP cohort, relapse occurred in the 3–52 months range (median: 17 months) after transplantation [6], further emphasizing the need to identify relapses early so that therapeutic measures can be instituted. The precondition for such monitoring is the availability of accredited laboratories that participate in external quality assurance. Results should be expressed on the International Scale and should indicate the molecular response [71]. Laboratories should ensure that their assay can detect disease down to a 4.5 log reduction below baseline [72]. If those requirements are met, monthly monitoring in the first year after HSCT and 2–3 monthly monitoring in the second year are recommended. The blood-based diagnostics should be supplemented by bone marrow aspiration on days +30, +60, +100, +180, and +365 as in acute leukemia. The clonal MRD marker in bone marrow by IgH or TCR rearrangements or by flow cytometry for CML-BP lymphoid phenotype can also be assessed if possible and available at the same time points. Additionally, in the post-transplant setting, a comparison of molecular markers could conceivably provide new insights into the disease biology of the CML-BP lymphoid phenotype.

#### pCML-WP recommendation

In addition to regular post-transplantation monitoring, *BCR::ABL1* transcript quantification is mandatory and should be closely performed, especially during the first two years after allogeneic HSCT. The consequence of detection is outlined in the next two subsections.

### Post-transplant TKI therapy and definition of molecular relapse

A survival benefit of TKI maintenance after allogeenic HSCT was observed in several retrospective and prospective trials that included adults and children allografted for Ph^+^ ALL or advanced phase CML [70, 73, 74]. However, the largest retrospective adult study exclusively enrolling patients with CML did not observe significant differences in leukemia-free survival and OS [75]. Nevertheless, the study by de Fillip et al. investigated all phases of CML and excluded patients with early molecular relapse (before day +100). The pCML-WP felt that the role of TKI maintenance in CML-BP should be to prevent early relapse.

The high rate of relapse in pediatric CML-BP after stem cell transplantation despite prior blast reduction indicates that the graft-versus-leukemia effect is not sufficient for sustained remission in every case [6]. Post-transplant TKI treatment is therefore recommended under certain conditions.

In principle, two treatment approaches can be pursued: a prophylactic approach after sufficient engraftment, or a preemptive intervention in cases of recurring or increasing *BCR::ABL1-*positive MRD. The study by Pfeifer et al. compared the two approaches in a randomized setting. That study enrolled 54 adult patients with Ph^+^ ALL or CML-BP lymphoid phenotype [70]. All received imatinib either after sufficient engraftment (prophylactic approach) or after a single detection of the *BCR::ABL1* transcript by quantitative PCR (preemptive approach). The incidence of molecular recurrence was significantly lower in the prophylactic than in the preemptive arm, but the long-term outcomes were not different in the groups. In the study population overall, early appearance of the MRD marker was found to be prognostic for poor leukemia-free survival. Thus, the prophylactic approach might be favored because early exposure to a TKI might control residual leukemia, avoiding molecular relapse before immunologic control arises through the graft-versus-leukemia effect. In that scenario, TKI toxicity becomes a major concern, and the criteria for starting a TKI should include sufficient hematologic recovery with adequate liver and kidney function, and controlled GvHD. Notably, in the study by Pfeifer et al., approximately 70% of the patients discontinued imatinib prematurely or required a dose reduction [70]. Thus, toxicity could potentially limit the duration of TKI administration after HSCT, potentially impairing the effectiveness of the prophylactic strategy [75]. Attention must also be paid to the fact that TKIs affect the pharmacokinetics of immunosuppressive therapy and vice versa. Furthermore, data exclusively for pediatric patients receiving the prophylactic approach are lacking.

Whether a restart of imatinib on day +56 after transplantation in the absence of contraindications is beneficial in pediatric Ph^+^ ALL is one of the study endpoints in the EsPhALL2017/Children’s Oncology Group AALL1631 trial (clinicaltrials.gov identifier NCT03007147), whose results were pending at the time of writing. Thus, no clear recommendation can be made for a prophylactic procedure.

**Fig. 2:**
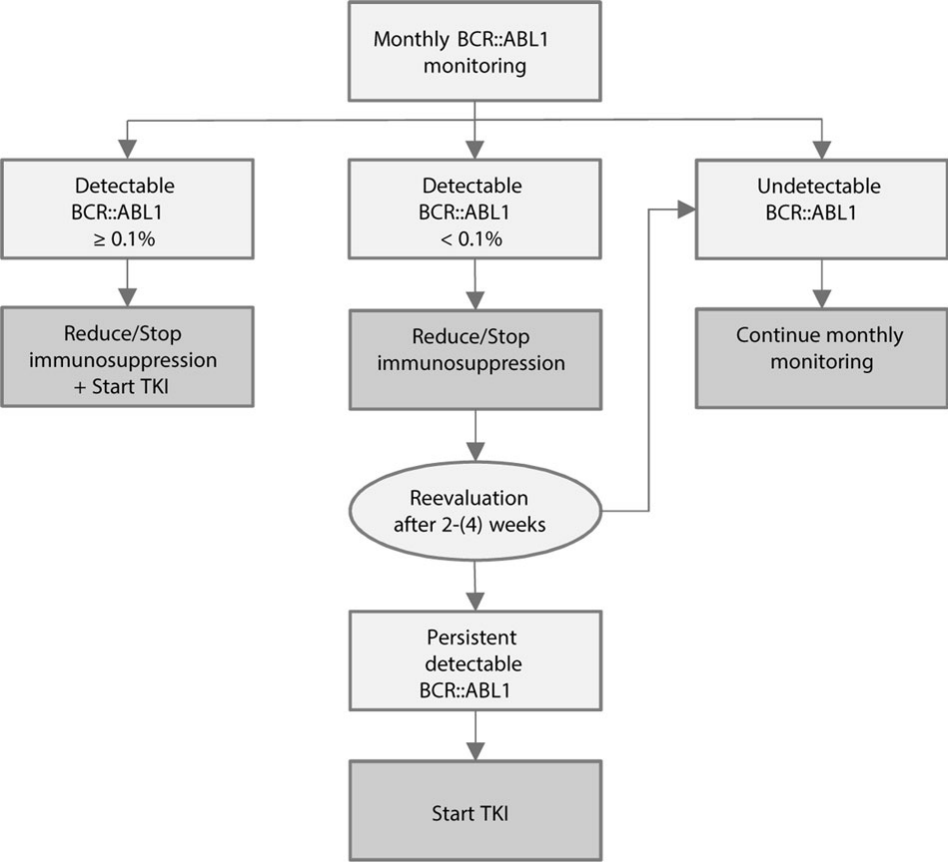
Approach in case of detectable *BCR::ABL1* transcripts after allogeneic hematopoietic stem cell transplantation (HSCT).

On the other hand, the preemptive approach requires the ability to use highly sensitive techniques for close monitoring of the *BCR::ABL1* transcript. In addition, a clear definition must be established for the restart of TKI therapy. The panel suggests defining molecular recurrence after HSCT as either loss of major molecular remission (MR 3, a *BCR::ABL1*/control gene ratio ≥0.1% on the International Scale) on a single measurement or detectable *BCR::ABL1* transcript at <0.1% on two consecutive measurements at a minimum interval of 2–4 weeks. If all requirements are fulfilled, the pCML-WP prefers a preemptive approach based on the current data for pediatric CML-BP. If the criteria are met, immunosuppression should be reduced depending on clinical status, harnessing the potential synergy of reduced immunosuppression to enhance the graft-versus-leukemia effect and TKI therapy initiation. A practical recommendation is presented in Figure 2. In the rare cases where a minor transcript type is detected, there is greater heterogeneity in terms of methodology for MRD measurement by real-time PCR. The EURO-MRD consortium guidance recommends a standardized approach for laboratories. In particular, standardization of primer/probe sets and the use of centrally produced plasmid calibrators reduced assay variability [76]. We therefore recommend that quantification be performed only in experienced laboratories that undergo regular quality assessments.

No comprehensive data regarding the minimal or optimal duration of TKI treatment after transplantation have been developed. In adults with Ph^+^ ALL, TKI administration for at least one year is assumed, with two years being recommended [77]. Maintaining deep molecular remission for at least two years was a criterion used in studies of imatinib discontinuation for treatment-free remission in both adult and pediatric patients with CML [78, 79]. If molecular detection of *BCR::ABL1* transcripts after HSCT has occurred, a similar approach would therefore be a reasonable recommendation. Concerning the choice of TKI, dasatinib appears to be more effective than imatinib in converting patients who are MRD-positive to negative status, but that conclusion is based on smaller retrospective cohort studies and a historical comparison in adult CML [80]. Based on the cited studies that used imatinib and the lack of data about toxicity with other TKIs after transplantation, imatinib would have to be considered the first choice unless resistance is demonstrated. For pediatric CML-BP, the panel recommends administering the same TKI both before and after HSCT.

#### pCML-WP recommendation

Provided that close monitoring of the *BCR::ABL1* transcript is implemented, a preemptive approach after HSCT is preferable. To allow for early intervention, the proposed molecular criteria for TKI restart are either loss of major molecular remission in a single sample or detectable *BCR::ABL1* transcripts at lower levels in two consecutive samples taken at least two weeks, but no more than 4 weeks, apart. TKI therapy should be continued for 2 years after stable deep molecular remission has been achieved. The same TKI agent that was administered before HSCT is recommended, provided that no new resistance mutations are detected.

### Treatment options for post-transplantation relapse

No relevant change in the reported relapse rates of 43–51% occurred after the introduction of TKIs in adult patients treated for advanced phase CML [55, 56]. Similar relapse rates of 30–45% are reported in children and adolescents with CML-BP who have undergone HSCT [5–7]. The initial approach to molecular relapse (discussed in the preceding subsection) is to restart TKI therapy and discontinue immunosuppressive therapy as soon as possible. If *BCR::ABL1* transcripts are repeatedly detectable at the same or increasing level, a diagnostic workup is suggested, including bone marrow morphology and cytogenetics to define the stage of relapse, and a tyrosine kinase domain mutation analysis. Although in most adult patients, the *BCR::ABL1* kinase mutation documented before transplantation persists, cases of clonal evolution in relapse after allograft are described [81]. Table 4 summarizes the diagnostic workup after relapse in CML-BP. Ponatinib would be the TKI of choice when a T315I mutation is present. In a case report, ponatinib was associated with a durable remission after molecular relapse in an adolescent patient with CML-BP [82]. The published pediatric experience with ponatinib is generally limited to smaller case series, but ponatinib has been associated with a molecular response in the 55–71% range in children with T315I mutations and children for whom several previous therapy lines have failed [83, 84]. Toxicities in children appear similar to those reported in adults, except for vascular events. Certainly, further information about safety in pediatric patients is needed. Ongoing phase I/II trials with or without chemotherapy are evaluating ponatinib in children (NCT04501614, NCT03934372). However, based on reported data at the time of writing, ponatinib should also be considered in pediatric patients after allogeneic HSCT for CML-BP lacking the T315I mutation, but with persistent or increasing molecular markers not responding to 2G-TKI treatment.

**Table 4:** Diagnostic work-up of relapse after allogeneic HSCT.

Diagnostics	
Bone Marrow	Morphology Blast % and promyelocytes % Flow cytometry and/or cytochemistry Cytogenetics (for Ph-positive and/or additional chromosomal aberrations) with a minimum of 15 metaphases analyzed Fluorescent-in-situ-hybridization (FISH) if marrow cytogenetics fails
Molecular genetics	Tyrosine kinase domain mutation analysis if *BCR::ABL1* transcript level >1%
Cerebrospinal Fluid	Cytology

Since the use of DLIs was first reported in the 1990s, strategies to treat relapse after HSCT have relied on their use. Historically, the graft-versus-leukemia effect is well described in adult CML, and DLI has been proven to induce durable remission [85, 86]. Nevertheless, data for CML-BP exclusively are sparse, and it is unclear whether the immunologic effects after allogeneic HSCT have a similar impact. Studies in adults have demonstrated synergy when DLIs are combined with TKIs [87, 88]. In a retrospective analysis of 215 CML patients who relapsed post-transplantation, outcomes after treatment with TKI combined with DLI, TKI only, and DLI only were examined [89]. In multivariate analysis, disease status before HSCT was significantly associated with OS, and OS was similar in patients who received only a TKI and in those who received a TKI plus DLI (*P* = 0.81). The authors concluded i) that despite the use of a TKI before transplantation, TKI salvage therapy provides a significant OS benefit for CML relapse after HSCT, and ii) that adding DLI to a TKI does not appear to improve OS. In another smaller series of 46 adult patients treated between 1993 and 2012 with either DLI (N = 28) or a TKI (*N* = 18) during a first CML relapse after HSCT (CML-CP, 37; CML-AP, 9), DLI was associated with inferior OS (hazard ratio, 37.4; 95% confidence interval, 2.2–625.4; *P* = 0.01), shorter failure-free survival (hazard ratio, 21.15; 95% confidence interval, 1.8–251; *P* = 0.02), a higher cumulative incidence of failure (hazard ratio, 19.5; 95% confidence interval, 1.6–236.5; *P* = 0.02), and an increased incidence of treatment-induced GvHD (68% vs. 6%; *P* = 0.001) [90]. A major concern is that DLI might induce severe GvHD, especially when administered early after HSCT. In a large series of 500 adult patients receiving treatment with DLI for CML relapse after HSCT (16% molecular, 30% cytogenetic, and 54% hematologic), the probability of survival in remission without secondary GvHD was highest (>50% at 5 years) when DLI was given beyond one year from HSCT for a molecular and/or cytogenetic CML relapse not preceded by chronic GvHD [91].

Given the lack of prospective studies in children to guide physicians in managing post-transplantation CML relapse, the risks and benefits of either TKI or DLI treatment should be evaluated carefully, and therapy should be tailored for each patient. For relapses presenting in advanced phase, the curative therapeutic approach would be a second remission induction and allogeneic HSCT. No data suggesting an “ideal” approach have been developed, and experimental treatment must be considered. Possible alternative therapeutic strategies are outlined in the “future prospects” section.

#### pCML-WP recommendation

To minimize the risk for GvHD, relapse treatment is recommended to begin with a second- or third-generation TKI, with resort to DLI as a second-line approach only in patients who do not respond to a TKI. For patients with relapse in the advanced phase, a second allogeneic HSCT, if possible with donor change, after induction of another hematologic remission remains the only curative treatment option at the time of writing. Participation in clinical trials should be attempted where feasible.

### Future prospects

Reducing the blastic cell pool, achieving a second CP, and proceeding with allogeneic HSCT as soon as possible are the main treatment steps in CML-BP. Most pediatric patients reach the goal of a second remission with immunophenotype-adapted chemotherapy plus TKI treatment. In CML-BP lymphoid phenotype, individualization of the induction intensity can be achieved with parallel monitoring of clone-specific IgH/TCR rearrangement markers and *BCR::ABL1* transcripts. Whether genetic risk features such as *IKZF1* deletions, which are associated with unfavorable outcomes in *BCR::ABL1*-positive ALL, also play a role in CML-BP and should be considered in therapy stratification has not been investigated [28].

To reduce chemotherapy exposure in patients with lymphoid malignancies, novel immunotherapeutic interventions are being intensively pursued and are increasingly advancing to the first line of therapy in current clinical trials. The bi-specific anti-CD3/CD19 monoclonal antibody blinatumomab has been associated with remission in patients with *BCR*::*ABL1-*positive ALL when given in combination with dasatinib and glucocorticoids [92]. In adult patients, four cases of CML-BP lymphoid phenotype treated with blinatumomab (three in combination with ponatinib, one in combination with dasatinib) have been reported [93, 94]. Rapid and deep remission occurred in all four patients, two proceeded to allogeneic HSCT, and all were alive after a follow-up of 3–8 months. Of two patients treated with a combination of inotuzumab ozogamicin and bosutinib, one responded [95]. Only a few children with CML-BP received blinatumomab under specific conditions. Further investigation is needed to confirm the role of immunotherapy (bispecific antibodies like blinatumomab, antibody–drug conjugates like inotuzumab ozogamicin, CAR-T cells) in pediatric patients with CML-BP B- lymphoid phenotype. However, this experience will expand significantly in the coming years with its wider use in common childhood ALL, making it a valuable alternative to chemotherapy, particularly for young patients.

No data on chimeric antigen receptor T cells in pediatric CML-BP have yet been developed. Conceptually, these cells represent an interesting alternative therapy in refractory cases with progenitor B lymphoid CML-BP [96]. However, the currently available CD19-directed chimeric antigen receptor T cells as well as specific antibodies target only B-lymphoblasts and presumably have little effect on the progenitor cell population of the *BCR*::*ABL1-*positive clone. There is, however a case report on an adult patient with lymphoid BP-CML harboring T315I mutation who achieved complete molecular remission and returned to chronic phase by anti-CD19 CAR-T therapy. It seemed that anti-CD19 CAR-T therapy cleared T315I mutation by eliminating CD19+ cell clones, which made the patient re-sensitize to the dasatinib [97]. Although CD26 has been identified as a leukemic stem cell marker in CML, its simultaneous expression in healthy stem cells probably excludes it as a possible target, at least for current constructs [98, 99].

Asciminib, a new inhibitor targeting the myristoyl pocket of the ABL1 kinase, has emerged as a promising agent for patients whose previous therapies have failed [100]. As the early-phase clinical trials included only a few individuals in AP, experience with asciminib in the advanced phase of CML is limited and has not been systematically evaluated [100, 101]. A pediatric study exploring the safety of asciminib in children has been initiated (NCT04925479). However, only patients in chronic phase are included here. Thus, experience regarding the role of asciminib in the blast phase will only emerge in the coming years.

A key question to be addressed in the future is the prognostic value of MRD level before transplantation. Extended acquisition and registration of molecular characteristics such as the presence of driver mutations should assist in the development of prognostic risk scores that can be used for therapy decision-making.

To summarize, children and adolescents with CML-BP benefit from early diagnosis, comprehensive characterization, and close monitoring. Data from the treatment of this rare entity should be collected in an international CML-BP registry. Treatment according to the recommendations presented here could contribute to improving knowledge and outcome in this critical disease entity.

## Supplementary information

Supplementary Figure 1.

## References

[1] Druker BJ, Guilhot F, O’Brien SG, Gathmann I, Kantarjian H, Gattermann N (2006). Five-year follow-up of patients receiving imatinib for chronic myeloid leukemia. N Engl J Med..

[2] Bower H, Björkholm M, Dickman PW, Höglund M, Lambert PC, Andersson TM (2016). Life expectancy of patients with chronic myeloid leukemia approaches the life expectancy of the general population. J Clin Oncol.

[3] Hehlmann R (2020). Chronic myeloid leukemia in 2020. Hemasphere.

[4] Hijiya N, Schultz KR, Metzler M, Millot F, Suttorp M (2016). Pediatric chronic myeloid leukemia is a unique disease that requires a different approach. Blood.

[5] Meyran D, Petit A, Guilhot J, Suttorp M, Sedlacek P, De Bont E (2020). Lymphoblastic predominance of blastic phase in children with chronic myeloid leukaemia treated with imatinib: A report from the I-CML-Ped Study. Eur J Cancer.

[6] Sembill S, Göhring G, Schirmer E, Lutterloh F, Suttorp M, Metzler M (2021). Paediatric chronic myeloid leukaemia presenting in de novo or secondary blast phase - a comparison of clinical and genetic characteristics. Br J Haematol.

[7] Millot F, Maledon N, Guilhot J, Güneş AM, Kalwak K, Suttorp M (2019). Favourable outcome of de novo advanced phases of childhood chronic myeloid leukaemia. Eur J Cancer.

[8] de la Fuente J, Baruchel A, Biondi A, de Bont E, Dresse MF, Suttorp M (2014). Managing children with chronic myeloid leukaemia (CML): recommendations for the management of CML in children and young people up to the age of 18 years. Br J Haematol.

[9] Hijiya N, Suttorp M (2019). How I treat chronic myeloid leukemia in children and adolescents. Blood.

[10] Hochhaus A, Baccarani M, Silver RT, Schiffer C, Apperley JF, Cervantes F (2020). European LeukemiaNet 2020 recommendations for treating chronic myeloid leukemia. Leukemia.

[11] Khoury JD, Solary E, Abla O, Akkari Y, Alaggio R, Apperley JF (2022). The 5th edition of the World Health Organization Classification of Haematolymphoid Tumours: Myeloid and Histiocytic/Dendritic Neoplasms. Leukemia.

[12] Arber DA, Orazi A, Hasserjian RP, Borowitz MJ, Calvo KR, Kvasnicka HM (2022). International consensus classification of myeloid neoplasms and acute leukemias: integrating morphologic, clinical, and genomic data. Blood.

[13] Lauseker M, Bachl K, Turkina A, Faber E, Prejzner W, Olsson-Stromberg U (2019). Prognosis of patients with chronic myeloid leukemia presenting in advanced phase is defined mainly by blast count, but also by age, chromosomal aberrations and hemoglobin. Am J Hematol.

[14] Suttorp M, Millot F, Sembill S, Deutsch H, Metzler M (2021). Definition, epidemiology, pathophysiology, and essential criteria for diagnosis of pediatric chronic myeloid leukemia. Cancers (Basel).

[15] Raanani P, Trakhtenbrot L, Rechavi G, Rosenthal E, Avigdor A, Brok-Simoni F (2005). Philadelphia-chromosome-positive T-lymphoblastic leukemia: acute leukemia or chronic myelogenous leukemia blastic crisis. Acta Haematol.

[16] Jain P, Kantarjian H, Jabbour E, Kanagal-Shamanna R, Patel K, Pierce S (2017). Clinical characteristics of Philadelphia positive T-cell lymphoid leukemias-(De novo and blast phase CML). Am J Hematol.

[17] Baccarani M, Castagnetti F, Gugliotta G, Rosti G, Soverini S, Albeer A (2019). The proportion of different BCR-ABL1 transcript types in chronic myeloid leukemia. An international overview. Leukemia.

[18] Hochhaus A, Reiter A, Skladny H, Melo JV, Sick C, Berger U (1996). A novel BCR-ABL fusion gene (e6a2) in a patient with Philadelphia chromosome-negative chronic myelogenous leukemia. Blood.

[19] Soverini S, Abruzzese E, Bocchia M, Bonifacio M, Galimberti S, Gozzini A (2019). Next-generation sequencing for BCR-ABL1 kinase domain mutation testing in patients with chronic myeloid leukemia: a position paper. J Hematol Oncol.

[20] Behrens YL, Schienke A, Davenport C, Lentes J, Tauscher M, Steinemann D (2021). BCR-ABL1 positive AML or CML in blast crisis? A pediatric case report with inv(3) and t(9;22) in the initial clone. Cancer Genet.

[21] Arber DA, Orazi A, Hasserjian R, Thiele J, Borowitz MJ, Le Beau MM (2016). The 2016 revision to the World Health Organization classification of myeloid neoplasms and acute leukemia. Blood.

[22] Nacheva EP, Grace CD, Brazma D, Gancheva K, Howard-Reeves J, Rai L (2013). Does BCR/ABL1 positive acute myeloid leukaemia exist?. Br J Haematol.

[23] Konoplev S, Yin CC, Kornblau SM, Kantarjian HM, Konopleva M, Andreeff M (2013). Molecular characterization of de novo Philadelphia chromosome-positive acute myeloid leukemia. Leuk Lymphoma.

[24] Suttorp M, Schulze P, Glauche I, Göhring G, von Neuhoff N, Metzler M (2018). Front-line imatinib treatment in children and adolescents with chronic myeloid leukemia: results from a phase III trial. Leukemia.

[25] Wang W, Hu Z (2022). Leukocytosis with left-shifted myeloid maturation in a peripheral blood specimen: a clue to the lymphoid blast phase of CML. Blood.

[26] Biondi A, Gandemer V, De Lorenzo P, Cario G, Campbell M, Castor A (2018). Imatinib treatment of paediatric Philadelphia chromosome-positive acute lymphoblastic leukaemia (EsPhALL2010): a prospective, intergroup, open-label, single-arm clinical trial. Lancet Haematol.

[27] Branford S, Kim DDH, Apperley JF, Eide CA, Mustjoki S, Ong ST (2019). Laying the foundation for genomically-based risk assessment in chronic myeloid leukemia. Leukemia.

[28] van der Veer A, Zaliova M, Mottadelli F, De Lorenzo P, Te Kronnie G, Harrison CJ (2014). IKZF1 status as a prognostic feature in BCR-ABL1-positive childhood ALL. Blood.

[29] Kolenova A, Maloney KW, Hunger SP (2016). Philadelphia chromosome-positive acute lymphoblastic leukemia or chronic myeloid leukemia in lymphoid blast crisis. J Pediatr Hematol Oncol.

[30] Zuna J, Hovorkova L, Krotka J, Koehrmann A, Bardini M, Winkowska L (2022). Minimal residual disease in BCR::ABL1-positive acute lymphoblastic leukemia: different significance in typical ALL and in CML-like disease. Leukemia.

[31] Hovorkova L, Zaliova M, Venn NC, Bleckmann K, Trkova M, Potuckova E (2017). Monitoring of childhood ALL using BCR-ABL1 genomic breakpoints identifies a subgroup with CML-like biology. Blood.

[32] Balducci E, Loosveld M, Rahal I, Boudjarane J, Alazard E, Missirian C (2018). Interphase FISH for BCR-ABL1 rearrangement on neutrophils: A decisive tool to discriminate a lymphoid blast crisis of chronic myeloid leukemia from a de novo BCR-ABL1 positive acute lymphoblastic leukemia. Hematol Oncol.

[33] Kamoda Y, Izumi K, Iioka F, Akasaka T, Nakamura F, Kishimori C (2016). Philadelphia chromosome-positive acute lymphoblastic leukemia is separated into two subgroups associated with survival by BCR-ABL fluorescence in situ hybridization of segmented cell nuclei: report from a single institution. Acta Haematol.

[34] Copland M (2022). Treatment of blast phase chronic myeloid leukaemia: A rare and challenging entity. Br J Haematol.

[35] Senapati J, Jabbour E, Kantarjian H, Short NJ. Pathogenesis and management of accelerated and blast phases of chronic myeloid leukemia. Leukemia. 2022. 10.1038/s41375-022-01736-5 Online ahead of print. 10.1038/s41375-022-01736-5 36309558

[36] Jain P, Kantarjian HM, Ghorab A, Sasaki K, Jabbour EJ, Nogueras Gonzalez G (2017). Prognostic factors and survival outcomes in patients with chronic myeloid leukemia in blast phase in the tyrosine kinase inhibitor era: Cohort study of 477 patients. Cancer..

[37] Branford S, Wang P, Yeung DT, Thomson D, Purins A, Wadham C (2018). Integrative genomic analysis reveals cancer-associated mutations at diagnosis of CML in patients with high-risk disease. Blood.

[38] Ochi Y, Yoshida K, Huang YJ, Kuo MC, Nannya Y, Sasaki K (2021). Clonal evolution and clinical implications of genetic abnormalities in blastic transformation of chronic myeloid leukaemia. Nat Commun.

[39] Ernst T, Busch M, Rinke J, Ernst J, Haferlach C, Beck JF (2018). Frequent ASXL1 mutations in children and young adults with chronic myeloid leukemia. Leukemia.

[40] Shah NP, Bhatia R, Altman JK, Amaya M, Begna KH, Berman E (2022). NCCN clinical practice guidelines in oncology, chronic myeloid leukemia V1.2023. Natl Compr Cancer Netw (NCCN).

[41] Strati P, Kantarjian H, Thomas D, O'Brien S, Konoplev S, Jorgensen JL (2014). HCVAD plus imatinib or dasatinib in lymphoid blastic phase chronic myeloid leukemia. Cancer.

[42] Benjamini O, Dumlao TL, Kantarjian H, O'Brien S, Garcia-Manero G, Faderl S (2014). Phase II trial of hyper CVAD and dasatinib in patients with relapsed Philadelphia chromosome positive acute lymphoblastic leukemia or blast phase chronic myeloid leukemia. Am J Hematol.

[43] Copland M, Slade D, McIlroy G, Horne G, Byrne JL, Rothwell K (2022). Ponatinib with fludarabine, cytarabine, idarubicin, and granulocyte colony-stimulating factor chemotherapy for patients with blast-phase chronic myeloid leukaemia (MATCHPOINT): a single-arm, multicentre, phase 1/2 trial. Lancet Haematol.

[44] Shen S, Chen X, Cai J, Yu J, Gao J, Hu S (2020). Effect of dasatinib vs imatinib in the treatment of pediatric philadelphia chromosome-positive acute lymphoblastic leukemia: a randomized clinical trial. JAMA Oncol.

[45] Zwaan CM, Rizzari C, Mechinaud F, Lancaster DL, Lehrnbecher T, van der Velden VH (2013). Dasatinib in children and adolescents with relapsed or refractory leukemia: results of the CA180-018 phase I dose-escalation study of the Innovative Therapies for Children with Cancer Consortium. J Clin Oncol.

[46] Slayton WB, Schultz KR, Kairalla JA, Devidas M, Mi X, Pulsipher MA (2018). Dasatinib plus intensive chemotherapy in children, adolescents, and young adults with philadelphia chromosome-positive acute lymphoblastic leukemia: results of children’s oncology group trial AALL0622. J Clin Oncol.

[47] Porkka K, Koskenvesa P, Lundán T, Rimpiläinen J, Mustjoki S, Smykla R (2008). Dasatinib crosses the blood-brain barrier and is an efficient therapy for central nervous system Philadelphia chromosome-positive leukemia. Blood.

[48] Chiba A, Toya T, Mizuno H, Tokushige J, Nakamura F, Nakazaki K (2018). Chronic myelogenous leukemia presenting with central nervous system infiltration, successfully treated with central nervous system-directed chemotherapy followed by allogeneic stem cell transplantation. Int J Hematol.

[49] Radhika N, Minakshi M, Rajesh M, Manas BR, Deepak, Kumar M (2011). Central nervous system blast crisis in chronic myeloid leukemia on imatinib mesylate therapy: report of two cases. Indian J Hematol Blood Transfus.

[50] Matsuda M, Morita Y, Shimada T, Miyatake J, Hirase C, Tanaka M (2005). Extramedullary blast crisis derived from 2 different clones in the central nervous system and neck during complete cytogenetic remission of chronic myelogenous leukemia treated with imatinib mesylate. Int J Hematol.

[51] Deau B, Nicolini FE, Guilhot J, Huguet F, Guerci A, Legros L (2011). The addition of daunorubicin to imatinib mesylate in combination with cytarabine improves the response rate and the survival of patients with myeloid blast crisis chronic myelogenous leukemia (AFR01 study). Leuk Res.

[52] Hrusak O, de Haas V, Stancikova J, Vakrmanova B, Janotova I, Mejstrikova E (2018). International cooperative study identifies treatment strategy in childhood ambiguous lineage leukemia. Blood.

[53] Hehlmann R, Voskanyan A, Lauseker M, Pfirrmann M, Kalmanti L, Rinaldetti S (2020). High-risk additional chromosomal abnormalities at low blast counts herald death by CML. Leukemia.

[54] Chen Z, Medeiros LJ, Kantajian HM, Zheng L, Gong Z, Patel KP (2017). Differential depth of treatment response required for optimal outcome in patients with blast phase versus chronic phase of chronic myeloid leukemia. Blood Cancer J.

[55] Niederwieser C, Morozova E, Zubarovskaya L, Zabelina T, Klyuchnikov E, Janson D (2021). Risk factors for outcome after allogeneic stem cell transplantation in patients with advanced phase CML. Bone Marrow Transpl.

[56] Radujkovic A, Dietrich S, Blok HJ, Nagler A, Ayuk F, Finke J (2019). Allogeneic stem cell transplantation for blast crisis chronic myeloid leukemia in the era of tyrosine kinase inhibitors: a retrospective study by the EBMT chronic malignancies working party. Biol Blood Marrow Transpl.

[57] Peters C, Schrappe M, von Stackelberg A, Schrauder A, Bader P, Ebell W (2015). Stem-cell transplantation in children with acute lymphoblastic leukemia: A prospective international multicenter trial comparing sibling donors with matched unrelated donors-The ALL-SCT-BFM-2003 trial. J Clin Oncol.

[58] Suttorp M, Claviez A, Bader P, Peters C, Gadner H, Ebell W (2009). Allogeneic stem cell transplantation for pediatric and adolescent patients with CML: results from the prospective trial CML-paed I. Klin Padiatr.

[59] Chaudhury S, Sparapani R, Hu ZH, Nishihori T, Abdel-Azim H, Malone A (2016). Outcomes of allogeneic hematopoietic cell transplantation in children and young adults with chronic myeloid leukemia: a CIBMTR cohort analysis. Biol Blood Marrow Transpl.

[60] Hafez HA, Abdallah A, Hammad M, Hamdy N, Yassin D, Salem S (2020). Outcomes of allogenic hematopoietic cell transplantation for childhood chronic myeloid leukemia: Single-center experience. Pediatr Transpl.

[61] Shimada H, Tanizawa A, Kondo T, Muramatsu H, Yasui M, Tojo A (2018). Sequential use of second-generation tyrosine kinase inhibitors following imatinib therapy in pediatric chronic myeloid leukemia: A report from the Japanese Pediatric Leukemia/Lymphoma Study Group. Blood.

[62] Zheng C, Zhu X, Tang B, Zhang X, Zhang L, Geng L (2018). Transplants of unrelated cord blood or sibling allogeneic peripheral blood stem cells/bone marrow in adolescent and young adults with chronic myeloid leukemia: comparable outcomes but better chronic GVHD-free and relapse-free survival among survivors with cord blood. Oncotarget.

[63] Chatterjee G, Rastogi N, Thakkar D, Kapoor R, Sharma A, Yadav SP (2021). Successful haploidentical stem cell transplant with posttransplant cyclophosphamide for isolated central nervous system blast crisis in a child with chronic myeloid leukemia. J Pediatr Hematol Oncol.

[64] Trujillo ÁM, Karduss AJ, Suarez G, Pérez R, Ruiz G, Cardona A (2021). Haploidentical hematopoietic stem cell transplantation with post-transplantation cyclophosphamide in children with high-risk leukemia using a reduced-intensity conditioning regimen and peripheral blood as the stem cell source. Transpl Cell Ther.

[65] Berger M, Lanino E, Cesaro S, Zecca M, Vassallo E, Faraci M (2016). Feasibility and outcome of haploidentical hematopoietic stem cell transplantation with post-transplant high-dose cyclophosphamide for children and adolescents with hematologic malignancies: an AIEOP-GITMO retrospective multicenter study. Biol Blood Marrow Transpl.

[66] Barrett AJ, Ito S (2015). The role of stem cell transplantation for chronic myelogenous leukemia in the 21st century. Blood..

[67] Shulman DS, Lee MA, Lehmann LE, Margossian SP (2016). Outcomes following bone marrow transplantation in children with accelerated phase or blast crisis chronic myelogenous leukemia in the era of tyrosine kinase inhibitors. J Pediatr Hematol Oncol.

[68] Peters C, Dalle JH, Locatelli F, Poetschger U, Sedlacek P, Buechner J (2021). Total body irradiation or chemotherapy conditioning in childhood ALL: a multinational, randomized, noninferiority phase III study. J Clin Oncol.

[69] Barrett J (2003). Allogeneic stem cell transplantation for chronic myeloid leukemia. Semin Hematol.

[70] Pfeifer H, Wassmann B, Bethge W, Dengler J, Bornhäuser M, Stadler M (2013). Randomized comparison of prophylactic and minimal residual disease-triggered imatinib after allogeneic stem cell transplantation for BCR-ABL1-positive acute lymphoblastic leukemia. Leukemia..

[71] Branford S, Fletcher L, Cross NC, Müller MC, Hochhaus A, Kim DW (2008). Desirable performance characteristics for BCR-ABL measurement on an international reporting scale to allow consistent interpretation of individual patient response and comparison of response rates between clinical trials. Blood.

[72] Branford S (2016). Molecular monitoring in chronic myeloid leukemia-how low can you go?. Hematol Am Soc Hematol Educ Program.

[73] Brissot E, Labopin M, Beckers MM, Socié G, Rambaldi A, Volin L (2015). Tyrosine kinase inhibitors improve long-term outcome of allogeneic hematopoietic stem cell transplantation for adult patients with Philadelphia chromosome positive acute lymphoblastic leukemia. Haematologica.

[74] Chen H, Liu KY, Xu LP, Liu DH, Chen YH, Zhao XY (2012). Administration of imatinib after allogeneic hematopoietic stem cell transplantation may improve disease-free survival for patients with Philadelphia chromosome-positive acute lymphobla stic leukemia. J Hematol Oncol.

[75] DeFilipp Z, Ancheta R, Liu Y, Hu ZH, Gale RP, Snyder D (2020). Maintenance tyrosine kinase inhibitors following allogeneic hematopoietic stem cell transplantation for chronic myelogenous leukemia: a center for international blood and marrow transplant research study. Biol Blood Marrow Transplant.

[76] Pfeifer H, Cazzaniga G, van der Velden VHJ, Cayuela JM, Schäfer B, Spinelli O (2019). Standardisation and consensus guidelines for minimal residual disease assessment in Philadelphia-positive acute lymphoblastic leukemia (Ph + ALL) by real-time quantitative reverse transcriptase PCR of e1a2 BCR-ABL1. Leukemia.

[77] Saini N, Marin D, Ledesma C, Delgado R, Rondon G, Popat UR (2020). Impact of TKIs post-allogeneic hematopoietic cell transplantation in Philadelphia chromosome-positive ALL. Blood.

[78] Mahon FX, Réa D, Guilhot J, Guilhot F, Huguet F, Nicolini F (2010). Discontinuation of imatinib in patients with chronic myeloid leukaemia who have maintained complete molecular remission for at least 2 years: the prospective, multicentre Stop Imatinib (STIM) trial. Lancet Oncol.

[79] de Bruijn CMA, Millot F, Suttorp M, Borisevich M, Brons P, Lausen B (2019). Discontinuation of imatinib in children with chronic myeloid leukaemia in sustained deep molecular remission: results of the STOP IMAPED study. Br J Haematol.

[80] Warraich Z, Tenneti P, Thai T, Hubben A, Amin H, McBride A (2020). Relapse prevention with tyrosine kinase inhibitors after allogeneic transplantation for philadelphia chromosome-positive acute lymphoblast leukemia: a systematic review. Biol Blood Marrow Transpl.

[81] Egan DN, Beppu L, Radich JP (2015). Patients with Philadelphia-positive leukemia with BCR-ABL kinase mutations before allogeneic transplantation predominantly relapse with the same mutation. Biol Blood Marrow Transpl.

[82] Lee JW, Yoo JW, Kim S, Jang PS, Chung NG, Cho B (2021). Efficacy of ponatinib prior to and after allogeneic hematopoietic stem cell transplantation in an adolescent with chronic myeloid leukemia in blast phase. Blood Res.

[83] Millot F, Suttorp M, Versluys AB, Kalwak K, Nelken B, Ducassou S (2020). Ponatinib in childhood Philadelphia chromosome-positive leukaemias: an international registry of childhood chronic myeloid leukaemia study. Eur J Cancer.

[84] Rossoff J, Huynh V, Rau RE, Macy ME, Sulis ML, Schultz KR (2020). Experience with ponatinib in paediatric patients with leukaemia. Br J Haematol.

[85] Kolb HJ, Mittermüller J, Clemm C, Holler E, Ledderose G, Brehm G (1990). Donor leukocyte transfusions for treatment of recurrent chronic myelogenous leukemia in marrow transplant patients. Blood.

[86] Collins RH, Shpilberg O, Drobyski WR, Porter DL, Giralt S, Champlin R (1997). Donor leukocyte infusions in 140 patients with relapsed malignancy after allogeneic bone marrow transplantation. J Clin Oncol.

[87] Zeidner JF, Zahurak M, Rosner GL, Gocke CD, Jones RJ, Smith BD (2015). The evolution of treatment strategies for patients with chronic myeloid leukemia relapsing after allogeneic bone marrow transplant: can tyrosine kinase inhibitors replace donor lymphocyte infusions?. Leuk Lymphoma.

[88] Savani BN, Montero A, Kurlander R, Childs R, Hensel N, Barrett AJ (2005). Imatinib synergizes with donor lymphocyte infusions to achieve rapid molecular remission of CML relapsing after allogeneic stem cell transplantation. Bone Marrow Transpl.

[89] Schmidt S, Liu Y, Hu ZH, Williams KM, Lazarus HM, Vij R (2020). The role of donor lymphocyte infusion (DLI) in post-hematopoietic cell transplant (HCT) relapse for chronic myeloid leukemia (CML) in the tyrosine kinase inhibitor (TKI) era. Biol Blood Marrow Transpl.

[90] Shanavas M, Messner HA, Kamel-Reid S, Atenafu EG, Gupta V, Kuruvilla J (2014). A comparison of long-term outcomes of donor lymphocyte infusions and tyrosine kinase inhibitors in patients with relapsed CML after allogeneic hematopoietic cell transplantation. Clin Lymphoma Myeloma Leuk.

[91] Radujkovic A, Guglielmi C, Bergantini S, Iacobelli S, van Biezen A, Milojkovic D (2015). Donor lymphocyte infusions for chronic myeloid leukemia relapsing after allogeneic stem cell transplantation: may we predict graft-versus-leukemia without graft-versus-host disease?. Biol Blood Marrow Transpl.

[92] Foà R, Bassan R, Vitale A, Elia L, Piciocchi A, Puzzolo MC (2020). Dasatinib-blinatumomab for ph-positive acute lymphoblastic leukemia in adults. N Engl J Med.

[93] Assi R, Kantarjian H, Short NJ, Daver N, Takahashi K, Garcia-Manero G (2017). Safety and efficacy of blinatumomab in combination with a tyrosine kinase inhibitor for the treatment of relapsed philadelphia chromosome-positive leukemia. Clin Lymphoma Myeloma Leuk.

[94] Patel SA, Bledsoe JR, Higgins AW, Hutchinson L, Gerber JM (2021). Rapid and deep remission induced by blinatumomab for CD19-positive chronic myeloid leukemia in lymphoid blast phase. JCO Precis Oncol.

[95] Jain N, Maiti A, Ravandi F, Konopleva M, Daver N, Kadia T (2021). Inotuzumab ozogamicin with bosutinib for relapsed or refractory Philadelphia chromosome positive acute lymphoblastic leukemia or lymphoid blast phase of chronic myeloid leukemia. Am J Hematol.

[96] Sun K, Zhang X, Wang Z, Chen Y, Zhang L, Cheng W (2018). Allogeneic CAR-T cell therapy for treatment of relapse after Allo-HSCT in patients with refractory CML lymphoid blast crisis: significance of HLA matched donor/patient pair in the safety/efficacy of CAR-T cell therapy. Blood.

[97] Zhou L, Shi H, Shi W, Yang L, Zhang Y, Xu M (2019). Durable molecular remission in a lymphoid BP-CML patient harboring T315I mutation treated with anti-CD19 CAR-T therapy. Onco Targets Ther.

[98] Rahman K, Singh MK, Chandra D, Gupta R, Sarkar MK, Gupta P (2022). CD26 expression on circulating CD34+/CD38- progenitor population is a specific and reliable tool for the rapid flow cytometric diagnosis of chronic myeloid leukemia-A single-center validation study. Int J Lab Hematol.

[99] Zhou S, Zhu X, Shen N, Li Q, Wang N, You Y (2019). T cells expressing CD26-specific chimeric antigen receptors exhibit extensive self-antigen-driven fratricide. Immunopharmacol Immunotoxicol.

[100] Réa D, Mauro MJ, Boquimpani C, Minami Y, Lomaia E, Voloshin S (2021). A phase 3, open-label, randomized study of asciminib, a STAMP inhibitor, vs bosutinib in CML after 2 or more prior TKIs. Blood.

[101] Hughes TP, Mauro MJ, Cortes JE, Minami H, Rea D, DeAngelo DJ (2019). Asciminib in chronic myeloid leukemia after ABL kinase inhibitor failure. N Engl J Med.

