# Supplementary material for: Management of children and adolescents with chronic myeloid leukemia in blast phase: International pediatric CML expert panel recommendations
Source: Leukemia. 2023 Jan 27;37(3):505–17. doi: 10.1038/s41375-023-01822-2 (PMC9991904; doi:10.1038/s41375-023-01822-2)
Supplement: Supplementary file 1 — Supplementary Figure 1. [file 41375_2023_1822_MOESM1_ESM.docx]

**Suppl. Figure 1. Summary and grading of recommendations based on advice from the GRADE working group.**

| **Topic** | **Recommendations** | **GoR** |
| --- | --- | --- |
| Diagnosis of CML-BP | The authors recommend an extended diagnostic workup for pediatric patients who meet the criteria of CML-BP (according to table2). | 1C |
|  | Clinicians should integrate a *BCR::ABL1* mutation analysis by NGS as one of the first diagnostic steps | 1C |
| Discrimination from Ph+-ALL | In B-lymphoid CML-BP it is suggested to monitor the clonal disease marker of the blast-population (either by Flow-cytometry or IgH/TCR rearrangements) in parallel with *BCR::ABL1* transcripts | 2C |
| General treatment principles in CML-BP | Allogenic HSCT is strongly recommended for most children with CML-BP | 1B |
|  | Before HSCT, a lineage-appropriate induction therapy in combination with a TKI should be administered | 1C |
| Selection of TKI-therapy | The authors recommend the use of a 2G-TKI in de-novo CML-BP | 2B |
|  | In secondary CML-BP it is strongly recommended to change the TKI | 1B |
|  | If a resistance-mediating BCR::ABL1 kinase domain mutation is present, clinicians should select a sensitive TKI | 1B |
|  | Ponatinib is the TKI of choice if a *T315I* mutation is detected | 1B |
|  | Consider ponatinib in case of 2G-TKI failure | 2C |
| Treatment of CML-BP lymphoid phenotype | Induction therapy is recommended to follow the institutional standard in combination with a TKI | 1C |
|  | TKI should be added immediately in case of secondary CML-BP or as soon as the *BCR::ABL1* translocation is confirmed for *de-novo* CML-BP | 1C |
|  | The authors recommend intrathecal chemoprophylaxis as per standard ALL induction protocols and afterwards monthly as bridging to allogenic HSCT | 1C |
|  | In case of CNS involvement, a cranial boost prior to conditioning is suggested | 2C |

| Treatment of CML-BP myeloid phenotype | The authors recommend administering a first AML-induction block following national and institutional standards | | 1C |
| --- | --- | --- | --- |
|  | To avoid interactions clinicians should commence TKI therapy at the end of the induction treatment | | 1C |
|  | Intrathecal chemoprophylaxis as per standard pediatric AML induction protocols is recommended | | 1C |
| Treatment of CML-BP mixed phenotype | | Based on the experience with Ph-negative mixed phenotype acute leukemia, the authors suggest a combination of ALL induction with a TKI unless other characteristics indicate different therapeutic options | 2C |
| Timing of allogenic HSCT | | Allogenic HSCT should be performed as soon as possible after, as a minimal requirement, a hematologic remission (2^nd^ CP) has been achieved, ideally within 3 months | 1C |
| Donor and graft selection | | The recommended donor type is a MSD or a MUD compatible in at least 9 of 10 HLA loci | 1C |
|  | | The authors suggest considering alternative donor types if no matched donor is available | 2C |
| Conditioning regimen and GvHD prophylaxis | | Generally, a myeloablative conditioning regimen is preferred | 2B |
|  | | TBI-based conditioning regimen should be preferred for lymphoid immunophenotype and any patient with CNS involvement | 1C |
|  | | The authors suggest GvHD-prophylaxis with anti-thymocyte globulin irrespective of donor type | 2B |
|  | | If possible, an early reduction of post-transplant immunosuppressive therapy is suggested | 2B |
| Post-transplant transcript monitoring | | *BCR::ABL1* transcripts should be monitored closely, especially during the first two years after allogenic HSCT | 1C |
| Post-transplant TKI therapy | | TKI therapy should be started in case of either loss of major molecular remission in a single sample or detectable *BCR::ABL1* transcripts at lower levels in two consecutive samples taken at least two weeks, but no more than 4 weeks, apart | 2C |
|  | | The authors recommend continuing post-transplant TKI therapy for 2 years after stable deep molecular remission | 2C |
| Treatment of post-transplant relapse | | To minimize the risk for GvHD, relapse treatment is recommended to begin with a second- or third-generation TKI, with resort to DLI as a second-line approach only in patients who do not respond to a TKI | 2C |
|  | | For patients with relapse in the advanced phase, a second allogeneic HSCT after induction of a second hematologic remission remains the only curative treatment option at the time of writing. | 2C |

Strength of recommendation: 1=strong, 2=weak; quality of evidence: A=high, B=moderate, C=low
